# Investigating the Psychology of Financial Markets During COVID-19 Era: A Case Study of the US and European Markets

**DOI:** 10.3389/fpsyg.2020.01924

**Published:** 2020-09-03

**Authors:** Khurram Shehzad, Liu Xiaoxing, Muhammad Arif, Khaliq Ur Rehman, Muhammad Ilyas

**Affiliations:** ^1^School of Economics and Management, Southeast University, Nanjing, China; ^2^School of Economics and Finance, Xi’an Jiaotong University, Xi’an, China; ^3^School of Management, Wuhan University of Technology, Wuhan, China

**Keywords:** COVID-19, financial stability, financial markets psychology, sustainable development, global development, health crisis, economic crisis

## Abstract

The novel coronavirus (COVID-19) has imperatively shaken the behavior of the global financial markets. This study estimated the impact of COVID-19 on the behavior of the financial markets of Europe and the US. The results revealed that the returns of the S&P 500 index have been greatly affected by a lockdown in the US owing to COVID-19. However, the health crisis generated due to the novel coronavirus significantly decreased the stock returns of the Nasdaq Composite index. The results also showed that the economic crisis generated from the pandemic in Spain has had more impact on the IBEX 35 as compared to the health crisis itself. On the other hand, in the long-run, Italy’s stock markets are more affected by the health crisis as contrasted with the economic crisis, while, in the short-run, both lockdown conditions and economic instability lower the stock returns of FTSE MIB. The UK stock markets witnessed that in the short-run, deficiency of health management systems imperatively damaged the stock returns of the London Stock Exchange. The investigation revealed that deficiency of health systems and lockdown conditions have imperatively damaged the structure of financial markets, inferring that sustainable development of these nations is at risk due to COVID-19. The study suggested that governments should allocate more of their budget to the health sector to overcome a health crisis in the future.

## Introduction

Historically, countries affected by a pandemic or epidemic that have seen large loss of life also see an impact within the economy and in their financial markets; a specific example would be the spread of Ebola disease in 2013–2016, which caused a loss of 53 billion dollars in the US (Fernandes, 2020). However, the potential damages of the current virus are still largely unknown. Significantly, the Coronavirus (COVID-19) infection disease was first reported in Wuhan on December 31, 2019 and spread rapidly to almost the whole world within the next few months (Albulescu, 2020). Compared to Severe Acute Respiratory Syndrome (SARS), COVID-19 is more contagious, which has been indicated by a different fatality rate^1^. COVID-19 has infected 1,865,413 individuals, and 110,008 deaths had been documented throughout the globe until mid-April 2020 (Financial Times, 2020). In order to eradicate this pandemic, the World Health Organization (WHO) recommended maintaining social distance, which generated the severe lockdown situation on the globe (WHO, 2020). Accordingly, the economic circle in the whole world has been disturbed. Notably, the sale of Online Travel Agencies (OTAs), Airlines, and Hotels has unexpectedly declined (World Economic Forum, 2020). Even oil prices have nosedived due to the sudden outbreak of this pandemic (EIA, 2020). Due to globalization, the current coronavirus outbreak will aggravate the economic condition, which can pave the way toward financial meltdown (Huang et al., 2020). This pandemic has caused a severe psychological impact on the economy while agitating service industries and financial markets.

**FIGURE 1:**
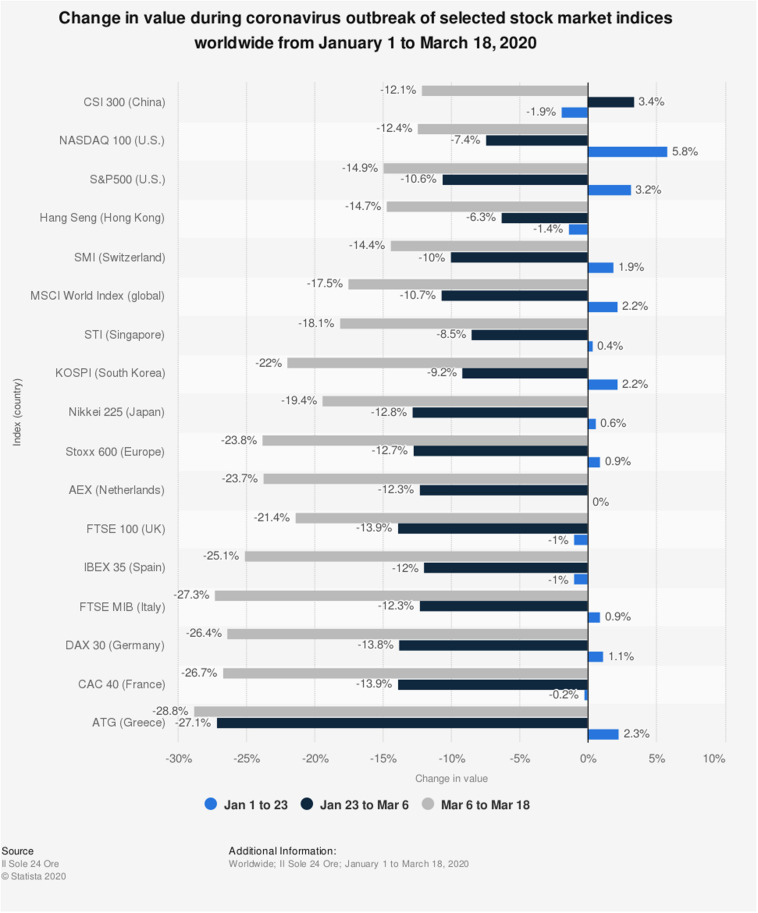
Deterioration in the stock markets owing to novel coronavirus.

Moreover, the coronavirus outbreak has severely affected the financial markets while declining the value of stock index up to 10% in 1 day (Daube, 2020). Surprisingly, news or specific events can fluctuate stock values (Shehzad and Sohail, 2018). Figure 1 revealed the market value in the European, American, Chinese, and Hong Kong markets from January 1, 2020–March 18, 2020. The period is divided into three rounds for comparison purposes; the first time-lagged is from January 1, 2020 to January 23, 2020, the second time-lagged is from January 23 to March 6, and the third time-lagged denoted the period of March 6, 2020 to March 18, 2020. The results showed that the market values of these indices significantly declined in the third time-lagged as COVID-19 approached its peak in western countries. Spain’s stock market share value was decreased by 27.3% in the third time-lagged. COVID-19 tremendously shrank Greece stock markets.

**FIGURE 2:**
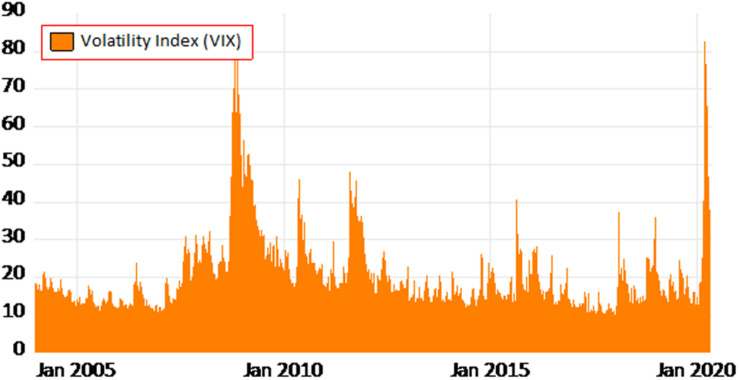
Variations in VIX due to Global Financial Crisis 2007–2009 and COVID-19 Crisis.

Moreover, Figure 2 illustrates the volatility index (VIX) from January 2005–March 2020. It denotes that the world has faced severe financial crises in 2007–2009. The stock markets collapsed, and the economies went through enormous pressure. A similar scenario has been built up during the global pandemic COVID-19. The stock market variance is at its highest for the second time after the Global Financial Crises (GFC).

In this regard, the novel coronavirus’s impact is also detrimental, and pandemic behavior is unknown, so its effect on the stock return can last longer. Relevant to this, the US stock volatility has been agitated due to the enormous spread of this pandemic resulting in severe economic crisis (Sharif et al., 2020). In such circumstances, it is quite significant to analyze coronavirus short-term and long-term impacts on the advanced countries’ stock return. This investigation argued that as the confirmed patients of novel coronavirus increase, the lockdown conditions becomes stricter, which lead to a significant economic crisis and shocked the economic stability of one’s nations. Moreover, deaths that befell as a result of novel coronavirus specified the deficiency of health facilities, which had caused a substantial health crisis. Particularly, this examination analyzed the effects of the economic and health crisis due to the novel coronavirus on the behavior of financial markets of the United States (US), Germany, United Kingdom (UK), Italy, and Spain for the period of February 10, 2020 to April 9, 2020.

This study elucidates the effects by executing a Non-linear Autoregressive Distributed lagged (NARDL) model. This investigation provides remarkable policies on how to handle the impacts of COVID-19 on the financial market and answers the momentous queries of the researchers, policymakers, Government officials, and academicians, e.g., firstly, does COVID-19 have a non-linear impact on financial markets’ behavior? Secondly, does the health crisis or economic instability generated by COVID-19 have a more significant impact on the S&P 500 index? Thirdly, does the Nasdaq Composite index or S&P 500 index respond more critically to the COVID-19 crisis? Fourthly, does the economic crisis generated by COVID-19 or the health crisis have a more significant impact on stock returns? Fifthly, how does this suspended circle of economy, due to lockdown, begin again? According to the author’s best knowledge, this is the first study that examines the asymmetrical impact of COVID-19 on the psychology of stock markets of mostly infected nations of the world. Firstly, this project ascertained the stationary level of study variables and discovered that all the variables are stationary at I(0) and I(1), and no one is stationary at I(2). Hence, a Non-linear version of the Autoregressive Distributed Lag (ARDL) model can be applied.

## Data and Methodology

### Data

This study utilized the daily data of the number of confirmed patients and deaths due to COVID-19 and stock markets of the US, Spain, Italy, Germany, and the UK, from the period of February 10, 2020–April 9, 2020. This study has taken the data from the database of Yahoo Finance and the European Center for Disease Control and Prevention. Further, this investigation analyzed the daily returns of IBEX 35, FTSE MIB, DAX 30, and London Stock Exchange (LSE) for the nomination of Spain, Italy, Germany, and the UK. However, S&P 500 and Nasdaq Composite Index represents the US.

### Methodology

This investigation utilized each stock market’s daily returns as the dependent variable and confirmed cases and deaths as the independent variable. The daily returns used in this study are computed as follows (Syllignakis and Kouretas, 2011; Shehzad and Sohail, 2018),

(1) R=ti(ln(CS/tiCS)t-1i)*100

here R_*t*__*i*_, CS_*t*_, and CS_*t–1*_ denote the return value at time t, closing price at time t, and previous day closing price at time t, for a stock market *i*, respectively. The linear association between these variables can be defined as;

(2) $$\Delta \,S\,P\,R_{t}=\beta_{0}+\beta_{1}\,U\,S\,C_{t}+\beta_{2}\,U\,S\,D_{t}+\varepsilon_{t}$$

(3) $$\Delta \,N\,S\,R_{t}=\beta_{0}+\beta_{1}\,U\,S\,C_{t}+\beta_{2}\,U\,S\,D_{t}+\varepsilon_{t}$$

(4) $$\Delta \,I\,B\,E\,R_{t}=\beta_{0}+\beta_{1}\,S\,P\,C_{t}+\beta_{2}\,S\,P\,D_{t}+\varepsilon_{t}$$

(5) $$\Delta \,F\,T\,M\,R_{t}=\beta_{0}+\beta_{1}\,I\,T\,L\,C_{t}+\beta_{2}\,I\,T\,L\,D_{t}+\varepsilon_{t}$$

(6) $$\Delta \,D\,A\,X\,R_{t}=\beta_{0}+\beta_{1}\,G\,E\,R\,C_{t}+\beta_{2}\,G\,E\,R\,D_{t}+\varepsilon_{t}$$

(7) $$\Delta \,L\,S\,E\,R_{t}=\beta_{0}+\beta_{1}\,U\,K\,C_{t}+\beta_{2}\,U\,K\,D_{t}+\varepsilon_{t}$$

where Δ signifies the first difference and β_0_β_1_β_2_ are the independent parameters. However, NSR, SPR, IBER, FTMR, DAXR, and LSER defines the return values of the Nasdaq Composite index, S&P 500, IBEX 35, FTSE MIB, DAX 30, and London Stock Exchange, respectively. Moreover, USC, SPC, ITLC, GERC, and UKC indicate the confirmed cases of COVID-19 in the US, Spain, Italy, Germany, and the UK, respectively. Further, USD, SPD, ITLD, GERD, and UKD refer to confirmed deaths due to COVID-19 in the US, Spain, Italy, Germany, and the UK, respectively.

To ascertain the short- and long-run asymmetries, this study employed the Non-Linear Autoregressive Distributed Lag (NARDL) model introduced by Shin et al. (2014). This model performs best with a small number of observations and can be applied on a mixed level of stationary data, i.e., I (0) and I(1). It evaluates the non-linearity and cointegration between variables in one equation; and shared the short-run variations to long-run asymmetries after taking the long-run parameters into account (Shin et al., 2014). The NARDL model is the extended form of the ARDL (Pesaran et al., 2001) model; hence, the full form of NARDL model for each stock market can be specified as,

$$\Delta \,S\,P\,R_{t}=\vartheta_{0}+\vartheta_{1}\,\sum_{i=1}^{p}\Delta \,S\,P\,R_{t-1}+\vartheta_{2\,a}\,\sum_{i=1}^{p}\Delta \,U\,S\,C_{t-1}^{+}+$$

$$\vartheta_{2\,b}\,\sum_{i=1}^{p}\Delta \,U\,S\,C_{t-1}^{-}\,\vartheta_{3\,a}\,\sum_{i=1}^{p}\Delta \,U\,S\,D_{t-1}^{+}+\vartheta_{3\,b}\,\sum_{i=1}^{p}\Delta \,U\,S\,D_{t-1}^{-}+\gamma_{1}$$

$$S\,P\,R_{t-i}+\gamma_{2\,a}\,U\,S\,C_{t-1}^{+}+\gamma_{2\,b}\,U\,S\,C_{t-1}^{-}+\gamma_{3\,a}\,U\,S\,D_{t-1}^{+}+\gamma_{3\,b}$$

(8) $$U\,S\,D_{t-1}^{-}+\mu_{t}$$

$$\Delta \,N\,S\,R_{t}=\vartheta_{0}+\vartheta_{1}\,\sum_{i=1}^{p}\Delta \,N\,S\,R_{t-1}+\vartheta_{2\,a}\,\sum_{i=1}^{p}\Delta \,U\,S\,C_{t-1}^{+}+$$

$$\vartheta_{2\,b}\,\sum_{i=1}^{p}\Delta \,U\,S\,C_{t-1}^{-}\,\vartheta_{3\,a}\,\sum_{i=1}^{p}\Delta \,U\,S\,D_{t-1}^{+}+\vartheta_{3\,b}\,\sum_{i=1}^{p}\Delta$$

$$U\,S\,D_{t-1}^{-}+\gamma_{1}\,N\,S\,R_{t-i}+\gamma_{2\,a}\,U\,S\,C_{t-1}^{+}+\gamma_{2\,b}\,U\,S\,C_{t-1}^{-}+\gamma_{3\,a}$$

(9) $$U\,S\,D_{t-1}^{+}+\gamma_{3\,b}\,U\,S\,D_{t-1}^{-}+\mu_{t}$$

$$\Delta \,I\,B\,E\,R_{t}=\vartheta_{0}+\vartheta_{1}\,\sum_{i=1}^{p}\Delta \,I\,B\,E\,R_{t-1}+\vartheta_{2\,a}\,\sum_{i=1}^{p}\Delta \,S\,P\,C_{t-1}^{+}+$$

$$\vartheta_{2\,b}\,\sum_{i=1}^{p}\Delta \,S\,P\,C_{t-1}^{-}\,\vartheta_{3\,a}\,\sum_{i=1}^{p}\Delta \,S\,P\,D_{t-1}^{+}+\vartheta_{3\,b}\,\sum_{i=1}^{p}\Delta$$

$$S\,P\,D_{t-1}^{-}+\gamma_{1}\,I\,B\,E\,R_{t-i}+\gamma_{2\,a}\,S\,P\,C_{t-1}^{+}+\gamma_{2\,b}\,S\,P\,C_{t-1}^{-}+\gamma_{3\,a}$$

(10) $$S\,P\,D_{t-1}^{+}+\gamma_{3\,b}\,S\,P\,D_{t-1}^{-}+\mu_{t}$$

$$\Delta \,F\,T\,M\,R_{t}=\vartheta_{0}+\vartheta_{1}\,\sum_{i=1}^{p}\Delta \,F\,T\,M\,R_{t-1}+\vartheta_{2\,a}\,\sum_{i=1}^{p}\Delta \,I\,T\,L\,C_{t-1}^{+}+$$

$$\vartheta_{2\,b}\,\sum_{i=1}^{p}\Delta \,I\,T\,L\,C_{t-1}^{-}\,\vartheta_{3\,a}\,\sum_{i=1}^{p}\Delta \,I\,T\,L\,D_{t-1}^{+}+\vartheta_{3\,b}\,\sum_{i=1}^{p}\Delta$$

$$I\,T\,L\,D_{t-1}^{-}+\gamma_{1}\,F\,T\,M\,R_{t-i}+\gamma_{2\,a}\,I\,T\,L\,C_{t-1}^{+}+\gamma_{2\,b}\,I\,T\,L\,C_{t-1}^{-}+\gamma_{3\,a}$$

(11) $$I\,T\,L\,D_{t-1}^{+}+\gamma_{3\,b}\,I\,T\,L\,D_{t-1}^{-}+\mu_{t}$$

$$\Delta \,D\,A\,X\,R_{t}=\vartheta_{0}+\vartheta_{1}\,\sum_{i=1}^{p}\Delta \,D\,A\,X\,R_{t-1}+\vartheta_{2\,a}\,\sum_{i=1}^{p}\Delta \,G\,E\,R\,C_{t-1}^{+}+$$

$$\vartheta_{2\,b}\,\sum_{i=1}^{p}\Delta \,G\,E\,R\,C_{t-1}^{-}+\vartheta_{3\,a}\,\sum_{i=1}^{p}\Delta \,G\,E\,R\,D_{t-1}^{+}+\vartheta_{3\,b}\,\sum_{i=1}^{p}\Delta$$

$$G\,E\,R\,D_{t-1}^{-}+\gamma_{1}\,D\,A\,X\,R_{t-i}+\gamma_{2\,a}\,G\,E\,R\,C_{t-1}^{+}+\gamma_{2\,b}\,G\,E\,R\,C_{t-1}^{-}+\gamma_{3\,a}$$

(12) $$G\,E\,R\,D_{t-1}^{+}+\gamma_{3\,b}\,G\,E\,R\,D_{t-1}^{-}+\mu_{t}$$

$$\Delta \,L\,S\,E\,R_{t}=\vartheta_{0}+\vartheta_{1}\,\sum_{i=1}^{p}\Delta \,L\,S\,E\,R_{t-1}+\vartheta_{2\,a}\,\sum_{i=1}^{p}\Delta \,U\,K\,C_{t-1}^{+}+$$

$$\vartheta_{2\,b}\,\sum_{i=1}^{p}\Delta \,U\,K\,C_{t-1}^{-}\,\vartheta_{3\,a}\,\sum_{i=1}^{p}\Delta \,U\,K\,D_{t-1}^{+}+\vartheta_{3\,b}\,\sum_{i=1}^{p}\Delta$$

$$U\,K\,D_{t-1}^{-}+\gamma_{1}\,L\,S\,E\,R_{t-i}+\gamma_{2\,a}\,U\,K\,C_{t-1}^{+}+\gamma_{2\,b}\,U\,K\,C_{t-1}^{-}+\gamma_{3\,a}$$

(13) $$U\,K\,D_{t-1}^{+}+\gamma_{3\,b}\,U\,K\,D_{t-1}^{-}+\mu_{t}$$

here ϑ_*0*_ϑ_1_,ϑ_2*a*_,ϑ_2*b*_ϑ_*3a*_and ϑ_*3b*_ are the short-run factors, while γ_*1*_, γ_*2a*_,  γ_*2b*_,γ_*3a*_,  and  γ_*3b*_ indicates the long-run parameters. Whereas, *i* symbolizes the number of lag used by the model based on Akaik Information Criterion and Schwarz Information Criterion. The above stated equations Eqs 8–13 assumed that confirmed number of cases and deaths due to COVID-19 have asymmetrical impact on stock returns.

Thus,  USC${}_{t-1}^{+}$,USD${}_{t-1}^{+}$,SPC${}_{t-1}^{+}$,  SPD${}_{t-1}^{+}$,ITLC${}_{t-1}^{+}$,  ITLD${}_{t-1}^{+}$,GERC${}_{t-1}^{+}$,  GERD${}_{t-1}^{+}$,  UKC${}_{t-1}^{+}$,  and  UKD${}_{t-1}^{+}$ designates a positive shock, though USC${}_{t-1}^{-}$, USD${}_{t-1}^{-}$, SPC${}_{t-1}^{-}$, SPD${}_{t-1}^{-}$, ITLC${}_{t-1}^{-}$, ITLD${}_{t-1}^{-}$,GERC${}_{t-1}^{-}$, GERD${}_{t-1}^{-}$,UKC${}_{t-1}^{-}$, and UKD${}_{t-1}^{-}$ implies a negative shock for each variable, and it is computed as follows,

(14) $$\rho_{t\,i}^{+}=\sum_{j=1}^{t}\Delta \,\rho_{j\,i}^{+}=\sum_{j=1}^{t}\text{max}\,\left(\Delta \,\rho_{\text{ji}},0\right)\,\left(14\right)$$

(15) $$\rho_{t\,i}^{-}=\sum_{j=1}^{t}\Delta \,\rho_{j\,i}^{-}=\sum_{j=1}^{t}\text{min}\,\left(\Delta \,\rho_{\text{ji}},0\right)\,\left(15\right)$$

here $\rho_{t\,i}^{+}$ delineates the positive shock in a variable *i* and *j* denotes the asymmetric distributive lag. The error correction form for these factors can be written as follows,

$$\Delta \,S\,P\,R_{t}=\vartheta_{0}+\vartheta_{1}\,\sum_{i=1}^{p}\Delta \,S\,P\,R_{t-1}+\vartheta_{2\,a}\,\sum_{i=1}^{p}\Delta \,U\,S\,C_{t-1}^{+}+$$

$$\vartheta_{2\,b}\,\sum_{i=1}^{p}\Delta \,U\,S\,C_{t-1}^{-}\,\vartheta_{3\,a}\,\sum_{i=1}^{p}\Delta \,U\,S\,D_{t-1}^{+}+\vartheta_{3\,b}\,\sum_{i=1}^{p}\Delta$$

(16) $$U\,S\,D_{t-1}^{-}+\phi_{1}\,E\,C\,T_{t-1}+\mu_{t}$$

$$\Delta \,N\,S\,R_{t}=\vartheta_{0}+\vartheta_{1}\,\sum_{i=1}^{p}\Delta \,N\,S\,R_{t-1}+\vartheta_{2\,a}\,\sum_{i=1}^{p}\Delta \,U\,S\,C_{t-1}^{+}+$$

$$\vartheta_{2\,b}\,\sum_{i=1}^{p}\Delta \,U\,S\,C_{t-1}^{-}\,\vartheta_{3\,a}\,\sum_{i=1}^{p}\Delta \,U\,S\,D_{t-1}^{+}+\vartheta_{3\,b}\,\sum_{i=1}^{p}\Delta$$

(17) $$U\,S\,D_{t-1}^{-}+\phi_{1}\,E\,C\,T_{t-1}+\mu_{t}$$

$$\Delta \,I\,B\,E\,R_{t}=\vartheta_{0}+\vartheta_{1}\,\sum_{i=1}^{p}\Delta \,I\,B\,E\,R_{t-1}+\vartheta_{2\,a}\,\sum_{i=1}^{p}\Delta \,S\,P\,C_{t-1}^{+}+$$

$$\vartheta_{2\,b}\,\sum_{i=1}^{p}\Delta \,S\,P\,C_{t-1}^{-}\,\vartheta_{3\,a}\,\sum_{i=1}^{p}\Delta \,S\,P\,D_{t-1}^{+}+\vartheta_{3\,b}\,\sum_{i=1}^{p}\Delta$$

(18) $$S\,P\,D_{t-1}^{-}+\phi_{1}\,E\,C\,T_{t-1}+\mu_{t}$$

$$\Delta \,F\,T\,M\,R_{t}=\vartheta_{0}+\vartheta_{1}\,\sum_{i=1}^{p}\Delta \,F\,T\,M\,R_{t-1}+\vartheta_{2\,a}\,\sum_{i=1}^{p}\Delta \,I\,T\,L\,C_{t-1}^{+}+$$

$$\vartheta_{2\,b}\,\sum_{i=1}^{p}\Delta \,I\,T\,L\,C_{t-1}^{-}\,\vartheta_{3\,a}\,\sum_{i=1}^{p}\Delta \,I\,T\,L\,D_{t-1}^{+}+\vartheta_{3\,b}\,\sum_{i=1}^{p}\Delta$$

(19) $$I\,T\,L\,D_{t-1}^{-}+\phi_{1}\,E\,C\,T_{t-1}+\mu_{t}$$

$$\Delta \,D\,A\,X\,R_{t}=\vartheta_{0}+\vartheta_{1}\,\sum_{i=1}^{p}\Delta \,D\,A\,X\,R_{t-1}+\vartheta_{2\,a}\,\sum_{i=1}^{p}\Delta \,G\,E\,R\,C_{t-1}^{+}+$$

$$\vartheta_{2\,b}\,\sum_{i=1}^{p}\Delta \,G\,E\,R\,C_{t-1}^{-}\,\vartheta_{3\,a}\,\sum_{i=1}^{p}\Delta \,G\,E\,R\,D_{t-1}^{+}+\vartheta_{3\,b}\,\sum_{i=1}^{p}\Delta$$

(20) $$G\,E\,R\,D_{t-1}^{-}+\phi_{1}\,E\,C\,T_{t-1}+\mu_{t}$$

$$\Delta \,L\,S\,E\,R_{t}=\vartheta_{0}+\vartheta_{1}\,\sum_{i=1}^{p}\Delta \,L\,S\,E\,R_{t-1}+\vartheta_{2\,a}\,\sum_{i=1}^{p}\Delta \,U\,K\,C_{t-1}^{+}+$$

$$\vartheta_{2\,b}\,\sum_{i=1}^{p}\Delta \,U\,K\,C_{t-1}^{-}\,\vartheta_{3\,a}\,\sum_{i=1}^{p}\Delta \,U\,K\,D_{t-1}^{+}+\vartheta_{3\,b}\,\sum_{i=1}^{p}\Delta$$

(21) $$U\,K\,D_{t-1}^{-}+\phi_{1}\,E\,C\,T_{t-1}+\mu_{t}$$

here *ECT*_*t–1*_ directs the error correction term. The long-run cointegration among variables is examined through the bound test approach of Pesaran et al. (2001). This method relies on *F-*test to evaluate the null hypothesis, H0: γ_1_ = γ_2*a*_ = + γ_3*a*_ = 0. For this purpose, Pesaran et al. (2001) has defined two bounds, i.e., upper and lower bound. If the estimated *F-*statistics are the higher than upper bound limit then the null hypothesis of no cointegration is rejected. However, if the projected value of *F-*test is lower than lower bound limit then null hypothesis cannot be rejected; besides, if the value of *F-*test remains between both limits then the results are not conclusive. At the end, this project employed the Wald test to verify the asymmetric long-run,  $\frac{\gamma_{2\,a}^{+}}{\gamma_{1}}=\frac{\gamma_{2\,b}^{-}}{\gamma_{1}},\frac{\gamma_{3\,a}^{+}}{\gamma_{1}}=\frac{\gamma_{3\,b}^{-}}{\gamma_{1}}\,a\,n\,d\,s\,h\,o\,r\,t-r\,u\,n\,\frac{\vartheta_{2\,a}^{+}}{\vartheta \,1}=\frac{\vartheta_{2\,b}^{-}}{\vartheta \,1},\frac{\vartheta_{3\,a}^{+}}{\vartheta \,1}$ = $\frac{\vartheta_{3\,b}^{-}}{\vartheta \,1}$ asymmetric relationship between study variables.

## Study Findings

The descriptive summary showed, in Table 1, during the COVID-19 era, the mean of stock returns is negative. Further, skewness values of these markets are also negative except LSER with high kurtosis, which predicts high chances of loss these days. Table 2 presents the results of the Augmented Dickey-Fuller (ADF) test (Dickey and Fuller, 1979) and Phillip Perron (PP) test (Phillips and Perron, 1988). It showed that the study variables have diverse stationary levels, i.e., I (0) and I (1), and no variable is cointegrated at I (2).

**TABLE 1:** Descriptive statistics.

	**IBER**	**SPD**	**SPC**	**LSER**	**UKC**	**UKD**	**SPR**	**USC**
Mean	−0.41737	147.2222	1463.792	−0.08997	621.2083	71.70833	−0.28187	3658.956
Skewness	−1.68003	1.687765	1.641313	0.027343	2.392073	3.081854	−0.35422	2.186996
Kurtosis	10.36907	4.166164	4.206693	4.903565	7.778953	11.75376	5.380136	6.389965
Jarque.Bera	196.7798	38.26244	36.69524	10.87966	137.1793	343.8589	17.47298	86.76708
Probability	0	0	0	0.00434	0	0	0.000161	0

	**USD**	**NR**	**GERC**	**GERD**	**DAXR**	**ITLD**	**ITLC**	**FTMR**

Mean	109.3088	−0.18523	1446.396	30.54717	−0.42086	174.9577	1365.254	−0.40245
Skewness	2.927378	−0.53985	1.120857	2.230936	−0.65869	1.232889	1.058817	−2.31595
Kurtosis	10.43888	5.59924	2.573906	7.120135	7.135106	2.868864	2.472667	14.04434
Jarque.Bera	253.9095	22.44518	11.49843	81.45175	41.59309	18.03773	14.08892	424.3192
Probability	0	0.000013	0.003185	0	0	0.000121	0.000872	0

*Source: Author’s calculation.*

**TABLE 2:** Unit root test.

	**ADF *F-*statistics**		**PP *F-*statistics**	
**Variable**	**Level**	**1st Diff.**	**Level**	**1st Diff.**
DAXR	−6.893713***	−8.54426***	−7.057616***	−37.2059****
GERC	−1.293963	−3.969976**	−0.971366	−14.51473***
GERD	−5.935247***	−4.359161***	3.147762	−6.393867***
FTMR	−4.631659	−11.55646***	−9.451458***	−30.1619***
ITLD	−1.127702	−4.174362***	−0.400067	−11.35456***
ITLC	−1.300231	−5.849862**	−0.488626	−8.372171***
IBER	−4.27056	−9.985616***	−9.446574***	−63.65801***
SPD	−1.506727	−10.32241***	0.159422	−10.09496***
SPC	4.552953	−7.028486***	−0.374729	−7.97616***
LSER	−8.775255***	−8.591517***	−8.768878***	−47.87539***
UKC	−7.816325***	−1.578091	2.746256	−9.61778***
UKD	7.429147***	−12.36282***	2.571441	−12.31254***
SPR	−12.89506***	−6.022076***	−12.13962***	−37.8073***
USC	−6.847753***	−5.567572**	4.310638**	−7.46537***
USD	3.138569	−5.097646**	7.384682***	−5.006879***
NSR	−13.66594***	−13.40744***	−12.64235***	−34.28961***

*, ** and *** denote 10, 5, and 1% level of significance, respectively. Source: Author’s calculation.

### Bound Test Cointegration Fallouts

The outcomes of the Bound test displayed in Table 3 disclosed significant statistical evidence of a long-term association between study variables for each equation. The *F-*statistics for joint significance of lagged level parameters are stated as 63.02, 39.11, 5.4, 5.1, 38.2, and 14.03 for Eqs 1–6, respectively. These values surpass the upper bound limits of Shin et al. (2014), implying that long-run cointegration exists among economic crisis, health crisis, and financial markets of each equation.

**TABLE 3:** Results of *F-*bound test.

**Test statistic**	**Value**	**Signif.**	**I(0)**	**I(1)**	**Test statistics**	**Value**	**Signif.**	**I(0)**	**I(1)**
SPR		10%	2.2	3.09	FTMR		10%	2.2	3.09
*F-*statistic	63.02410	5%	2.56	3.49	*F-*statistic	5.165881	5%	2.56	3.49
K	4	2.5%	2.88	3.87	K	4	2.5%	2.88	3.87
		1%	3.29	4.37			1%	3.29	4.37
NSR		10%	2.2	3.09	DAXR		10%	2.2	3.09
*F-*statistic	39.11238	5%	2.56	3.49	*F-*statistic	38.23325	5%	2.56	3.49
K	4	2.5%	2.88	3.87	K	4	2.5%	2.88	3.87
		1%	3.29	4.37			1%	3.29	4.37
IBER		10%	2.2	3.09	LSER		10%	2.2	3.09
*F-*statistic	5.490181	5%	2.56	3.49	*F-*statistic	14.03259	5%	2.56	3.49
K	4	2.5%	2.88	3.87	K	4	2.5%	2.88	3.87
		1%	3.29	4.37			1%	3.29	4.37

*Source: Author’s calculation.*

### Long- and Short-Run Asymmetric Cointegration Reckoning

Table 4 exhibited the upshots of the Wald-test. The statistics particularized that USC and USD have a non-linear association with SPR. However, USC revealed short-run asymmetric affiliation with NSR. Besides, SPD caused an asymmetric influence on IBER for both long-term and short-term periods, while SPC possessed short-run asymmetry only. Additionally, the results of FTMR designated that ITLD has a long-run asymmetric relationship with it. The Wald-statistics of GERC enlightened that it has both long and short-run non-linear impacts on DAXR, but GERD has a short-term non-linear affiliation with DAXR. Moreover, UKC denotes a long-run asymmetric liaison with LSER. Hence, the existence of non-linearity in the study variables led us to choose the NARDL model for computing long-and short-run factors.

**TABLE 4:** Wald test.

**Long-run asymmetry**		**Short-run asymmetry**		**Long-run asymmetry**		**Short-run asymmetry**	
SPR	*F-*stat		*F-*stat	FTMR	*F-*stat		*F-*stat
USC	8.093***	USC	6.224**	ITC	1.242605	ITC	0.2916
USD	4.779**	USD	6.815**	ITD	5.864**	ITD	0.1767
NSR				DAXR			
USC	1.574046	USC	4.228**	GERC	5.208**	GERC	17.24***
USD	2.589403	USD	1.609162	GERD	2.338933	GERD	18.87***
IBER				LSER			
SPC	1.569179	SPC	2.8616*	UKC	0.725822	UKC	0.640751
SPD	2.61007*	SPD	6.8974**	UKD	0.786146	UKD	0.718125

*, **, and *** denote 10, 5, and 1% level of significance, respectively. Source: Author’s calculation.

### Modeling NARDL Parameters

The non-linear fallouts of SPR, presented in Table 5, illustrated that expansion and contraction in USC brings a reduction and increase in SPR, respectively. It indicates that strict lockdown substantially decreases the market returns of the S&P 500 and vice versa. Further, a positive shock in USD caused a significant rise in SPR. In the short-run, lagged values specified that a negative shock in USC amplified SPR. Besides, all the lagged terms described that an uptick effect in USD improved the SPR. Also, a negative shock in USD and its second lag augmented the SPR, but the first and third lag represents the reverse impact. Furthermore, the negative and significant value of ECT_*t*__–__1_ stated that disequilibrium occurred in SPR today due to COVID-19 will adjust with the speed of 1.3 units on a subsequent day. The *R-*squared value indicated that the 91% volatility in SPR is owing to COVID-19.

**TABLE 5:** Asymmetric parameters of SPR.

**Variables**	**Short-term coefficients**	**Std. Error**	***t*-stat**	**Prob.**
D(USC_NEG)	−0.047867***	0.003539	−13.52394	0
D(USC_NEG(-1))	−0.061323***	0.003402	−18.02596	0
D(USC_NEG(-2))	−0.108895***	0.005569	−19.55339	0
D(USC_NEG(-3))	−0.072915***	0.003619	−20.14618	0
D(USD_POS)	0.713848***	0.036514	19.55011	0
D(USD_POS(-1))	0.23431***	0.014268	16.42276	0
D(USD_POS(-2))	0.194744***	0.01369	14.22562	0
D(USD_POS(-3))	0.334891***	0.029691	11.27907	0
D(USD_NEG)	−0.377433***	0.055371	−6.816426	0
D(USD_NEG(-1))	1.65514***	0.097958	16.89641	0
D(USD_NEG(-2))	−0.074692***	0.02737	−2.728986	0
D(USD_NEG(-3))	3.650475***	0.235321	15.51275	0
ECT_*t*__–__1_	−1.369525***	0.066813	−20.49782	0
*R-*Squared	0.912909			
Adjusted *R-*squared	0.892007			
Durbin-Watson stat	2.006439			

**Variables**	**Long-term Coefficients.**	**Std. Error**	***t*-stat**	**Prob.**

USC_POS	−0.004491***	0.001667	−2.694157	0.0099
USC_NEG	−0.054381***	0.014406	3.774912	0.0005
USD_POS	0.064436**	0.029631	2.174626	0.035
USD_NEG	−0.290139	0.338048	−0.858278	0.3953
C	0.181416	0.264851	0.684971	0.4969

*, **, and *** denote 10, 5, and 1% level of significance, respectively. Source: Author’s calculation.

The NSR model results exhibited in Table 6, particularized that both positive and negative shocks in USC escalate the NSR, while enlargement (reduction) in USD instigate decrease (increase) in NSR. It specifies that an upsurge in the health crisis in the US expressively distressed the Nasdaq Composite Index and vice versa. Nonetheless, in the short-run, a negative shock in USD and USC demonstrated an imperative decrease in NSR, whereas an expansion in USC lessens the NSR. The negative and significant ECT_*t*__–__1_ parameter stated that financial instability generated due to COVID-19 would be settled with a speed of 1.5 units on the next day. The *R-*square value signified that 81% of instability occurring in NSR is the result of COVID-19.

**TABLE 6:** Asymmetric parameters of NSR.

**Variables**	**Short-term coefficients**	**Std. Error**	***t*-stat**	**Prob.**
D(USD_NEG)	0.039324**	0.019503	2.016267	0.0487
D(USC_POS)	−0.002147***	0.000382	−5.622014	0
D(USC_NEG)	0.002319***	0.00069	3.360414	0.0014
D(USC_NEG(-1))	−0.008198***	0.001064	−7.703282	0
ECT_*t*__–__1_	−1.501702***	0.093855	−16.00026	0
*R-*Squared	0.812596			
Adjusted *R-*squared	0.800102			
Durbin-Watson stat	2.130245			

**Variables**	**Long-term coefficients**	**Std. Error**	***t*-stat**	**Prob.**

USD_POS	−0.013042***	0.004465	−2.920922	0.0051
USD_NEG	−0.026413	0.030567	−0.864107	0.3913
USC_POS	0.001013***	0.00034	2.977381	0.0043
USC_NEG	0.004258**	0.001923	2.214416	0.031
C	−0.525513*	0.274541	−1.914153	0.0608

*, **, and *** denote 10, 5, and 1% level of significance, respectively. *Source:* Author’s calculation.

The finding parameters of the IBER model (Table 7) identified that an assertive shock in SPD has an encouraging effect on IBER. Besides, adverse shocks remain insignificant. On the other hand, an escalation (decline) in SPC has a negative (positive) influence on IBER, implying that strict lockdown considerably caused the financial crisis in Spain for the long term period. In the short term period, an intensification in SPC, SPD, and a decline in SPC, SPD increased the IBER. The negative and significant value of ECT_*t*__–__1_ elucidated that today’s disequilibrium in IBER will adjust on the next day with a speed of 0.82 units. The coefficient of *R-*square stated that 72% of variations in IBER are owing to COVID-19.

**TABLE 7:** Asymmetric parameters of IBER.

**Variables**	**Short-term coefficients**	**Std. Error**	***t*-stat**	**Prob.**
D(IBER(-1))	−0.368944***	0.09522	−3.875	0.0003
D(SPD_POS)	0.018471*	0.009268	1.993	0.0512
D(SPD_NEG)	−0.026216**	0.010675	−2.456	0.0172
D(SPC_POS)	0.001511	0.000911	1.6591	0.1028
D(SPC_NEG)	−0.001832*	0.000966	−1.897	0.0631
D(SPC_NEG(-1))	0.00301***	0.000996	3.0316	0.0037
D(SPC_NEG(-2))	0.006948***	0.00129	5.3867	0
ECT_*t*__–__1_	−0.824331***	0.137511	−5.995	0
*R-*Squared	0.726141			
Adjusted *R-*squared	0.694191			
Durbin-Watson stat	2.154368			

**Variables**	**Long-term coefficients**	**Std. Error**	***t*-stat**	**Prob.**

SPD_POS	0.053991***	0.01765	3.0589	0.0034
SPD_NEG	0.023488	0.018017	1.3037	0.1978
SPC_POS	−0.006341***	0.001968	−3.222	0.0021
SPC_NEG	−0.005173**	0.001945	−2.659	0.0102
C	−0.636028	0.428132	−1.486	0.1431

*, **, and *** denote 10, 5, and 1% level of significance, respectively. *Source:* Author’s calculation.

Table 8 displayed the fallouts of the FTSE MIB stock market. The condition of COVID-19 on Italy’s stock markets exposed that rise (reduction) in ITLD diminished (amplified) the FTMR, inferring that heath crisis imperatively contributes to Italy’s financial instability for the long term period. In addition, growth in ITLC enlarged the FTMR imperatively. The sort-run condition in Italy denoted that first, second, and third lag values of FTMR harmed its own returns. Also, growth in ITLC has a negative impression on FTMR. Nonetheless, a negative shock in the second lag value of ITLC indicated an encouraging effect on FTMR. The consequences of ITLD symbolized that proliferation in ITLD possess negative sway on FTMER, but the first and second lag value have definite sway on it. Furthermore, a decline in ITLD has a direct influence on FTMR. The negative and significant figure of ECT_*t*__–__1_ itemized that FTMR will readjust the equilibrium with a speed of 0.95 units each day. The *R-*squared parameter denoted that 85% uncertainty generated in FTMR caused by COVID-19.

**TABLE 8:** Asymmetric parameters of FTMR.

**Variables**	**Short-term coefficients**	**Std. Error**	***t*-stat**	**Prob.**
D(FTMR(-1))	−0.27947**	0.138377	−2.019624	0.0493
D(FTMR(-2))	−0.154875	0.136743	−1.132605	0.2632
D(FTMR(-3))	−0.295127**	0.113398	−2.602569	0.0124
D(ITLC_POS)	−0.008711***	0.001397	−6.235569	0
D(ITLC_POS(-1))	−0.015154***	0.003264	−4.642728	0
D(ITLC_POS(-2))	−0.0047	0.002589	−1.573306	0.1225
D(ITLC_NEG)	−0.001115	0.001937	−0.575378	0.5678
D(ITLC_NEG(-1))	0.006885***	0.00198	3.477965	0.0011
D(ITLC_NEG(-2))	−0.003758**	0.001782	−2.109409	0.0404
D(ITLD_POS)	−0.06983***	0.014714	−4.745672	0
D(ITLD_POS(-1))	0.023801*	0.011962	1.989722	0.0526
D(ITLD_POS(-2))	0.029575***	0.010511	2.813763	0.0072
D(ITLD_NEG)	0.002565	0.012862	0.199404	0.8428
D(ITLD_NEG(-1))	0.038093**	0.015194	2.507186	0.0158
D(ITLD_NEG(-2))	0.04502***	0.013664	3.294772	0.0019
ECT_*t*__–__1_	−0.959373***	0.163657	−5.862111	0
*R-*Squared	0.851016			
Adjusted *R-*squared	0.807197			
Durbin-Watson stat	1.763297			

**Variables**	**Long-term coefficients**	**Std. Error**	***t*-stat**	**Prob.**

ITLC_POS	0.013317**	0.005649	2.357295	0.0227
ITLC_NEG	0.001321	0.002682	0.492464	0.6247
ITLD_POS	−0.109433**	0.053273	−2.054209	0.0457
ITLD_NEG	−0.048345**	0.020559	−2.351573	0.023
C	−0.500777	0.455916	−1.098396	0.2777

*, **, and *** denote 10%, 5% and 1% level of significance, respectively. *Source:* Author’s calculation.

The parameters of DAX 30 (Table 9) directed, in the long run, an upsurge (diminution) in GERD compacted (enlarge) the DAXR. Moreover, the decline in GERC also improves DAXR. These findings revealed that both lockdown and health crises are harmful to the stock markets of Germany. The short-term findings described that second, third, and fourth lag of positive shocks in GERC has a lessening impact on DAXR. However, a negative shock in GERC and other lag values except the first lag indicated an indirect association with DAXR. Additionally, positive shocks in GERD quantified mixed impact on DAXR, while a short term decline in GERD has negative, but first lag, second lag, and third lag values point out positive linkage with DAXR. These effects are because of instability generated due to COVID-19. The *R-*squared coefficient diagnosed that the 94.5% instability in DAXR is because of COVID-19.

**TABLE 9:** Asymmetric parameters of DAXR.

**Variables**	**Short-term coefficients**	**Std. Error**	***t*-stat**	**Prob.**
D(GERC_POS)	0.015476***	0.001209	12.80122	0
D(GERC_POS(-1))	0.012439***	0.001923	6.467043	0
D(GERC_POS(-2))	−0.088025***	0.005402	−16.29596	0
D(GERC_POS(-3))	−0.057992***	0.003854	−15.04828	0
D(GERC_POS(-4))	−0.097***	0.00647	−14.99249	0
D(GERC_NEG)	0.05202***	0.004721	11.11903	0
D(GERC_NEG(-1))	−0.067796***	0.004325	−15.67443	0
D(GERC_NEG(-2))	0.044564***	0.003649	12.21376	0
D(GERC_NEG(-3))	0.01327***	0.004158	3.191441	0.0041
D(GERC_NEG(-4))	0.183056***	0.01145	15.98705	0
D(GERD_POS)	−5.101477***	0.313432	−16.27619	0
D(GERD_POS(-1))	5.265597***	0.35462	14.84858	0
D(GERD_POS(-2))	−0.054459	0.100825	−0.540134	0.5943
D(GERD_POS(-3))	6.261756***	0.386781	16.18939	0
D(GERD_NEG)	−3.913296***	0.288936	−13.54383	0
D(GERD_NEG(-1))	30.64757***	1.850055	16.56576	0
D(GERD_NEG(-2))	23.76795***	1.466544	16.20677	0
D(GERD_NEG(-3))	19.4969***	1.21192	16.08761	0
ECT_*t*__–__1_	−0.497359***	0.029762	−16.71134	0
*R-*Squared	0.945788			
Adjusted *R-*squared	0.910937			
Durbin-Watson stat	1.667758			
Variables	Long-term Coefficients.	Std. Error	t-stat	Prob.
GERC_POS	0.016748	0.035958	0.465776	0.6458
GERC_NEG	−0.255912**	0.1228	−2.083972	0.0485
GERD_POS	−19.28734***	7.569148	−2.548151	0.018
GERD_NEG	−65.49979**	25.3728	−2.581496	0.0167
C	−2.440744**	0.93232	−2.617925	0.0154

*, **, and *** denote 10, 5, and 1% level of significance, respectively. *Source:* Author’s calculation.

**FIGURE 3:**
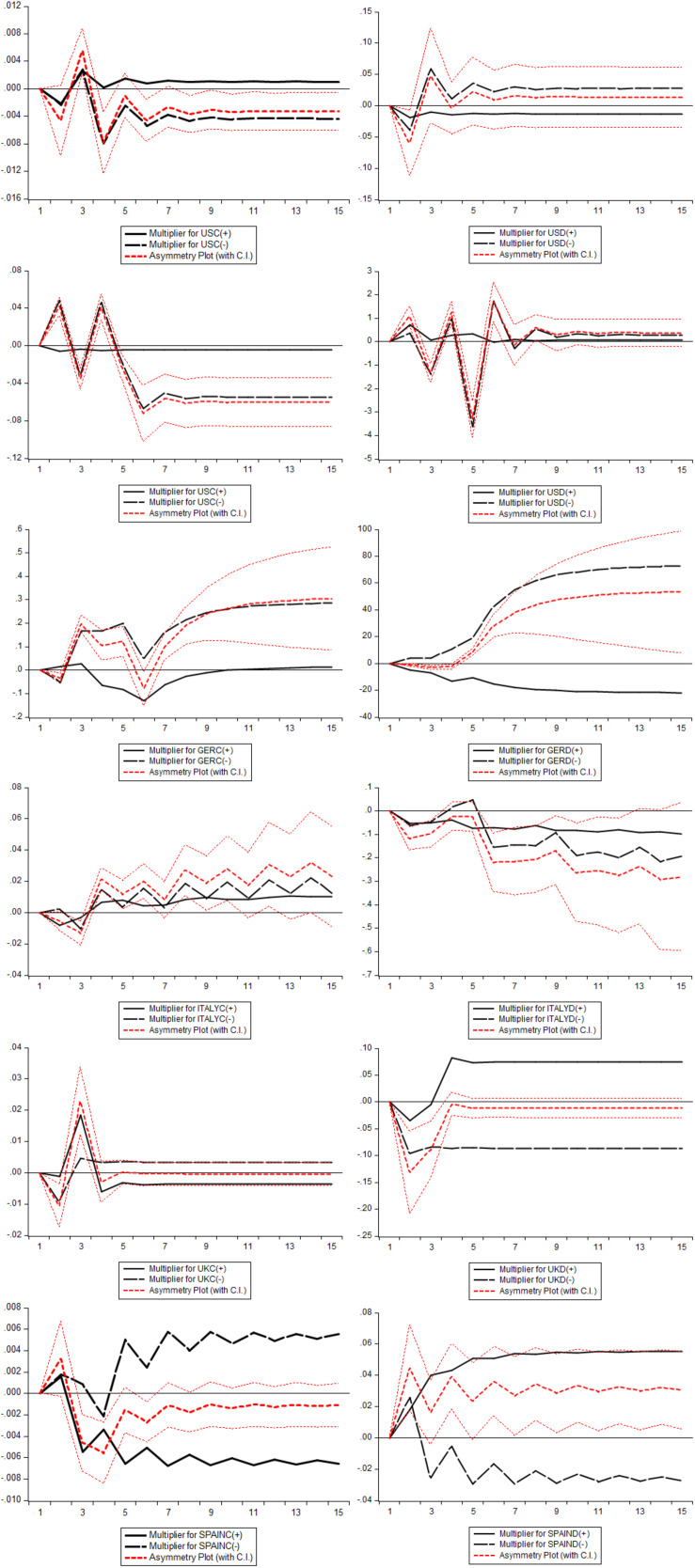
The dynamic multiplier plots.

Table 10 represents the results of the London Stock exchange. The concluding coefficients of the UK explored that augmentation in UKD improved the LSER, while diminution in UKD reduces the LSER in the long term period, although the growth of UKC brought a significant decrease in LSER. The short term period outlined that positive shocks in UKC reduced the LSER, but the first lag value of positive shock in UKD showed an alternate impression. Additionally, an upturn in UKD possesses a diminishing impact on LSER. These outcomes designated that lockdown and health crises generated due to COVID-19 negatively impact the financial markets of the UK for the short term period. The value of ECTt-1 showed that fluxes occurred today in LSER will get equilibrium with the speed of 1.1 units on the next day. The *R-*square value reported a 65% variation in LSER due to COVID-19. Figure 3 depicts the adjustment of asymmetric effect in the existing long-run equilibrium, once moved to a new long-run equilibrium as a result of positive and adverse shocks. The asymmetric plots denote the alliance of dynamic multipliers owing to positive and adverse shocks in S&P 500, DAX 30, FTSE MIB, Nasdaq Composite index, IBEX 35, and LSE markets. The outcomes revealed that these markets tremendously respond to the positive and negative shocks befallen due to COVID-19.

**TABLE 10:** Asymmetric parameters of LSER.

**Variables**	**Short-term coefficients**	**Std. Error**	***t*-stat**	**Prob.**
D(UKC_POS)	−0.00127	0.001661	−0.764723	0.4475
D(UKC_POS(-1))	0.022345***	0.003248	6.878603	0
D(UKC_NEG)	0.009267***	0.002137	4.337228	0.0001
D(UKD_POS)	−0.03503***	0.00746	−4.695883	0
D(UKD_POS(-1))	−0.090212***	0.014812	−6.090388	0
ECT_*t*__–__1_	−1.100754***	0.115104	−9.563147	0
*R-*Squared	0.651816			
Adjusted *R-*squared	0.624182			
Durbin-Watson stat	1.867108			

**Variables**	**Long-term coefficients**	**Std. Error**	***t*-stat**	**Prob.**

UKC_POS	−0.003609*	0.00191	−1.889866	0.0638
UKC_NEG	−0.003213	0.002112	−1.521044	0.1337
UKD_POS	0.075038**	0.02864	2.620078	0.0112
UKD_NEG	0.086022**	0.035056	2.453823	0.0172
C	−0.319529	0.353596	−0.903654	0.3699

*, **, and *** denote 10, 5, and 1% level of significance, respectively. *Source:* Author’s calculation.

### Diagnostic Parameters Evaluation

**FIGURE 4:**
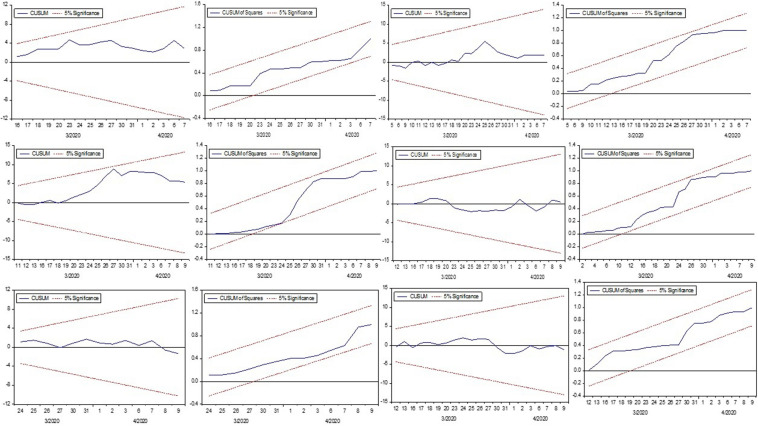
The CUSUM and CUSUMSQ graph.

The examination employed the Breusch and Pagan (2006) test to diagnose the Serial Correlation and Heteroskedasticity in each model’s residuals, Ramsey RESET technique, to ascertain functional misspecification, CUSUM, and CUSUMSQ (Ploberger and Kramer, 2006) test to define the reliability of parameters. The conclusions of these tests given in Table 11, nominated that there was no serial correlation and Heteroskedasticity in the residuals of each model. Besides, the plots of CUSUM and CUSUMSQ presented in Figure 4 remain within the 5% critical boundaries, inferring that coefficients of the NARDL model are stable.

**TABLE 11:** Diagnostic test results.

**Heteroskedasticity**	**Value**	**Serial correlation**	**Value**	**Heteroskedasticity**	**Value**	**Serial correlation**	**Value**
SR				FTMR			
*F-*statistic	1.321	*F-*statistic	0.5464	*F-*statistic	1.527	*F-*statistic	2.3644
Ramsey RESET		*F-*statistic	2.3623	Ramsey RESET		*F-*statistic	1.7672
NSR				DAXR			
Heteroskedasticity		Serial Correlation		Heteroskedasticity		Serial Correlation	
*F-*statistic	0.210	*F-*statistic	0.6362	*F-*statistic	0.264	*F-*statistic	0.8397
Ramsey RESET		*F-*statistic	0.1927	Ramsey RESET		*F-*statistic	0.2731
IBER				LSER			
Heteroskedasticity		Serial Correlation		Heteroskedasticity		Serial correlation	
*F-*statistic	0.929	*F-*statistic	1.4019	*F-*statistic	0.479	*F-*statistic	1.9939
Ramsey RESET		*F-*statistic	0.4698	Ramsey RESET		*F-*statistic	1.3502

*, **, and *** denote 10, 5, and 1% level of significance, respectively. *Source:* Author’s calculation.

## Conclusion

COVID-19 has imperatively shaken the economic indicators, primarily the financial markets of the globe. The number of patients and deaths as a result of COVID-19 are increasing day by day, and economic conditions have become entirely uncertain. Moreover, the financial markets of the world fronted a sudden crash in their market values. This investigation utilized the Non-linear Autoregressive Distributed Lag (NARDL) approach to estimate the non-linear impact of economic crisis and health crisis generated as a result of the COVID-19 pandemic on the behavior of financial markets of the most infected territories of Europe and the US. The analysis stated that the returns of the S&P 500 index are greatly affected by the economic crisis generated because of COVID-19 in the US. However, an accession in the health crisis, because of COVID-19, significantly decreased the Nasdaq Composite Index returns. These results confirmed that economic instability and the health management system have an imperative control over the financial markets’ behavior. The verdicts elaborated that the economic crisis produced as a result of lockdown circumstances in Spain has more impact on IBEX 35 as compared to the health crisis that resulted in Spain. In the long-run, Italy’s stock markets are more affected by the health crisis than the economic crisis, while, in the short-run, economic uncertainty also lowered the stock returns of FTSE MIB. Nevertheless, the health crisis in Germany pointing to a significant upsurge in the economic crisis intimated an unimportant impression on the FTSE 100 index. The stock markets of the UK recorded that, in the short-run, an upsurge in the health crisis imperatively damages the stock returns of the LSE index, but in the long run, this influence was left to reverse. Therefore, this research authenticated that the health crisis begun with COVID-19 has devised another global financial crisis, and almost every nation is fighting with these crises today. Moreover, the study disclosed that economic uncertainty generated by COVID-19 is highest in the German stock markets, while the S&P 500 index is on the second-ranking, and Italian stock markets are on the third-ranking. The study concluded that the health crisis and economic crisis ominously affected the stock markets of the globe. Consequently, a significant amount should be allocated in the budget to explore and prevent these pandemics in the future. Further, a complete lockdown strategy does not prove to be an excellent remedy as it harms the financial markets, so other strategies should be developed, i.e., smart/partial lockdown. These suggestions are essential for policymakers, Government officials, researchers, and the general public.

## Data Availability Statement

Publicly available datasets were analyzed in this study. This data can be found here: https://www.ecdc.europa.eu/en.

## Author Contributions

KS: conceptualization, analysis, and methodology. LX: supervision. MI: introduction. MA: results and discussion, and conclusion. KR: revising work, proofreading, and visualization. All authors contributed to the article and approved the submitted version.

## Conflict of Interest

The authors declare that the research was conducted in the absence of any commercial or financial relationships that could be construed as a potential conflict of interest.

## Footnotes

1 The SARS fatality rate was 11% while the fatality rate by novel coronavirus is 4% all around the globe.

## References

[B1] Albulescu C. (2020). *Coronavirus and Oil Price Crash.* Ithaca, NY: Cornell University.

[B2] Breusch T. S. Pagan A. R. (2006). A simple test for heteroscedasticity and random coefficient variation. *Econometrica* 47 1287–1294. 10.2307/1911963

[B3] Daube C. H. (2020). *The Corona Virus Stock Exchange Crash.* Kiel: Leibniz Information Centre for Economics.

[B4] Dickey D. Fuller W. (1979). Distribution of the estimators for autoregressive time series with a unit root. *J. Am. Stat. Assoc.* 74 427–431. 10.1080/01621459.1979.10482531

[B5] EIA (2020). *U.S. Energy Information Administration (EIA).* Available online at: https://www.eia.gov/ (accessed April 20, 2020).

[B6] Fernandes N. (2020). *Economic Effects of Coronavirus Outbreak (COVID-19) on the World Economy.* Pamplona: University of Navarra.

[B7] Financial Times (2020). *Coronavirus: US Death Toll Passes 16,000 Level | Financial Times.* Available online at: https://www.ft.com/content/55a136c0-05f1-39f4-b946-9e099dd05256 (accessed April 13, 2020).

[B8] Huang Y. Lin C. Wang P. Xu Z. (2020). *Saving China from the Coronavirus and Economic Meltdown: Experiences and Lessons.* London: SSRN.

[B9] Pesaran M. H. Shin Y. Smith R. J. (2001). Bounds testing approaches to the analysis of level relationships. *J. Appl. Econ.* 16 289–326. 10.1002/jae.616

[B10] Phillips P. C. B. Perron P. (1988). Testing for a unit root in time series regression. *Biometrika* 75 335–346. 10.1093/biomet/75.2.335

[B11] Ploberger W. Kramer W. (2006). The cusum test with ols residuals. *Econometrica* 60 271–285. 10.2307/2951597

[B12] Sharif A. Aloui C. Yarovaya L. (2020). COVID-19 pandemic, oil prices, stock market and policy uncertainty nexus in the US economy: fresh evidence from the wavelet-based approach. *Int. Rev. Finan. Anal.* 70:101496 10.1016/j.irfa.2020.101496 PMC7227524 38620230

[B13] Shehzad K. Sohail N. (2018). An evidence of calendar effects on the stock market of Pakistan?: a case study of (KSE-100 index). *NFC IEFR J. Eng. Sci. Res.* 6 46–56. 10.24081/nijesr.2018.1.0006

[B14] Shin Y. Yu B. Greenwood-Nimmo M. (2014). “Modelling asymmetric cointegration and dynamic multipliers in a non-linear ARDL framework,” in *Festschrift in Honor of Peter Schmidt*, eds Sickles R. Horrace W. C. (New York, NY: Springer), 281–314. 10.1007/978-1-4899-8008-3_9

[B15] Syllignakis M. N. Kouretas G. P. (2011). Dynamic correlation analysis of financial contagion: evidence from the Central and Eastern European markets. *Int. Rev. Econ. Finance.* 20 717–732. 10.1016/j.iref.2011.01.006

[B16] WHO (2020). *WHO Coronavirus Disease (COVID-19) Dashboard.* Geneva: WHO.

[B17] World Economic Forum (2020). *The Long Economic Reach of COVID-19 | World Economic Forum.* Available online at: https://www.weforum.org/agenda/2020/02/economic-toll-coronavirus-manufacturing-tourism-china-asia/ (accessed April 11, 2020).

